# Long rDNA amplicon sequencing of insect-infecting nephridiophagids reveals their affiliation to the Chytridiomycota and a potential to switch between hosts

**DOI:** 10.1038/s41598-020-79842-6

**Published:** 2021-01-11

**Authors:** Jürgen F. H. Strassert, Christian Wurzbacher, Vincent Hervé, Taraha Antany, Andreas Brune, Renate Radek

**Affiliations:** 1 Evolutionary Biology, Institute for Biology/Zoology, Free University of Berlin, Berlin, Germany; 2 Chair of Urban Water Systems Engineering, Technical University of Munich, Garching, Germany; 3 Research Group Insect Gut Microbiology and Symbiosis, Max Planck Institute for Terrestrial Microbiology, Marburg, Germany

**Keywords:** Ecology, Evolution, Microbiology

## Abstract

Nephridiophagids are unicellular eukaryotes that parasitize the Malpighian tubules of numerous insects. Their life cycle comprises multinucleate vegetative plasmodia that divide into oligonucleate and uninucleate cells, and sporogonial plasmodia that form uninucleate spores. Nephridiophagids are poor in morphological characteristics, and although they have been tentatively identified as early-branching fungi based on the SSU rRNA gene sequences of three species, their exact position within the fungal tree of live remained unclear. In this study, we describe two new species of nephridiophagids (*Nephridiophaga postici* and *Nephridiophaga javanicae*) from cockroaches. Using long-read sequencing of the nearly complete rDNA operon of numerous further species obtained from cockroaches and earwigs to improve the resolution of the phylogenetic analysis, we found a robust affiliation of nephridiophagids with the Chytridiomycota—a group of zoosporic fungi that comprises parasites of diverse host taxa, such as microphytes, plants, and amphibians. The presence of the same nephridiophagid species in two only distantly related cockroaches indicates that their host specificity is not as strict as generally assumed.

## Introduction

Insects are the most diverse group of all animals. So far, about one million species have been described and recent estimates for extant species range from 2.6 to 7.8 million^1,2^. They are globally distributed and impact human life at numerous levels. In agriculture, for instance, insects play a major role as both pollinators (e.g., honey bees) and pests that feed on crops (e.g., grasshoppers). Other pest insects live parasitic (e.g., lice) and/or transmit parasites and diseases (e.g., mosquitoes, cockroaches). Among the unicellular eukaryotes that infect insects, alveolates (apicomplexans, ciliates), amoeba, trypanosomes, and microsporidians are frequently found^3^. Nephridiophagids represent a further unicellular eukaryote group of insect parasites^4–7^. First discovered in honey bees (formal description of the genus *Nephridiophaga*)^5^, they are mainly known from cockroaches and beetles^8^. They infect the Malpighian tubules where especially the lumen can be densely colonised by different life cycle stages^9^. The life cycle of nephridiophagids consists of a vegetative phase with multinucleated plasmodia that multiply by division into oligonucleate and uninucleate cells, and a sporogenic phase with plasmodia that form uninucleate 5–10 μm long spores. Mature spores have a thick chitinous wall and are released with the feces of the host insects enabling infection of further individuals by oral uptake^9,10^.

The phylogenetic affiliation of nephridiophagids, which are poor in morphological characteristics, has been heavily debated and is far from being resolved^5,9,11–13^. Classifications of this taxon to diverse groups such as Microsporidia or Haplosporidia exemplify this controversy^11,13–15^. Only the molecular phylogenetic analysis of the small subunit ribosomal RNA (SSU rRNA) gene of one *Nephridiophaga* species (*N. blattellae*) in 2004 could shed light on the fungal nature of this parasite, although with only moderate statistical support^16^. Recently, the SSU rRNA gene sequence analysis of two further *Nephridiophaga* species (*N. blaberi*, *N. maderae*) confirmed the affiliation of nephridiophagids to the fungi but failed to safely assign them to any existing group^17^.

It is generally assumed that nephridiophagids are highly host-specific. Feeding of nephridiophagid spores from one cockroach species to another nephridiophage-free species did not lead to a successful transmission^9^. However, it needs to be noted that cockroaches donating and receiving nephridiophagids were fairly distantly related belonging to different families^9,18^. Whether the intimate association of host and parasite resulted in a cospeciation between the two partners, as it has been documented for other symbiotic relationships^19–23^, is currently unknown and awaits investigation.

In the present study, we screened putative nephridiophagid hosts for infections. Subsequently, we used long-read sequencing of the rDNA operon of various nephridiophagid species (two of them formally described here) in order to increase and analyse the phylogenetic signal for this enigmatic group, and to define its position in the fungal tree. We additionally inferred the molecular phylogeny of the host insects from their cytochrome *c* oxidase subunit II (COII) gene sequences, and—by comparing host and parasite phylogeny—discuss the potential for a cospeciation between the two partners.

## Results

### Morphology of *Nephridiophaga postici* sp. nov. and *Nephridiophaga javanicae* sp. nov.

**Figure 1:**
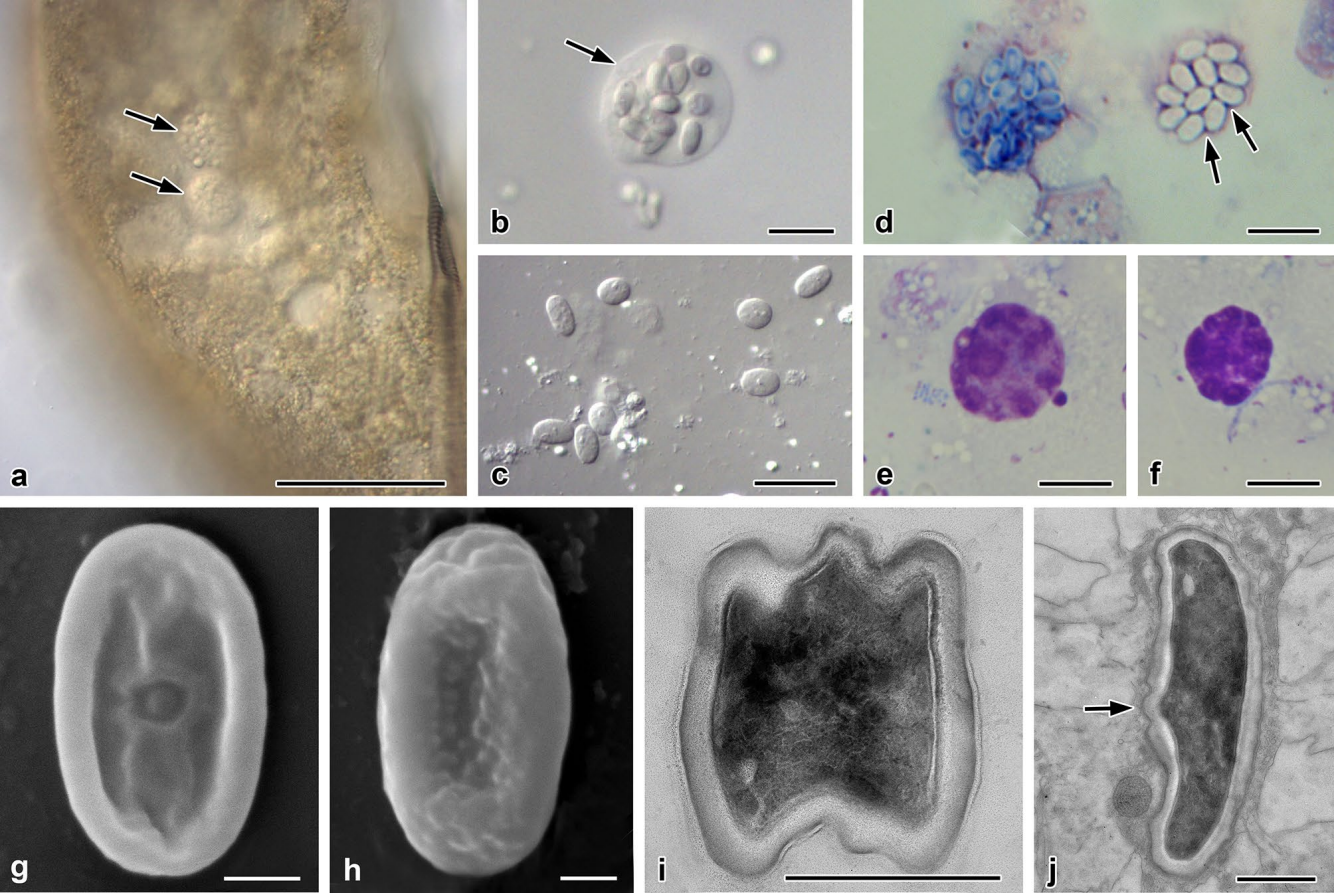
*Nephridiophaga postici* sp. nov. from *Eublaberus posticus*. (**a**) Infected Malpighian tubule containing sporogenic plasmodia (arrows). (**b**) Sporogenic plasmodium. Arrow points to plasma membrane of plasmodium. (**c**) Single spores. (**d**–**f**) Giemsa staining. (**d**) Left plasmodium with sporoblasts, right with mature spores. Arrows point to vegetative nuclei. (**e**,**f**) Vegetative plasmodia with stained nuclei. (**g**,**h**) Scanning electron micrographs of dorsal (**g**) and ventral (**h**) side of flattened, mature spores. (**i**,**j**) Ultra-thin sections. (**i**) Mature spore in cross-section. (**j**) Mature spore in longitudinal section. Arrow points to region of spore opening. Multilayered spore wall. Scale bars: (a) = 50 µm, (b–f) = 10 µm, (g–j) = 1 µm.

**Figure 2:**
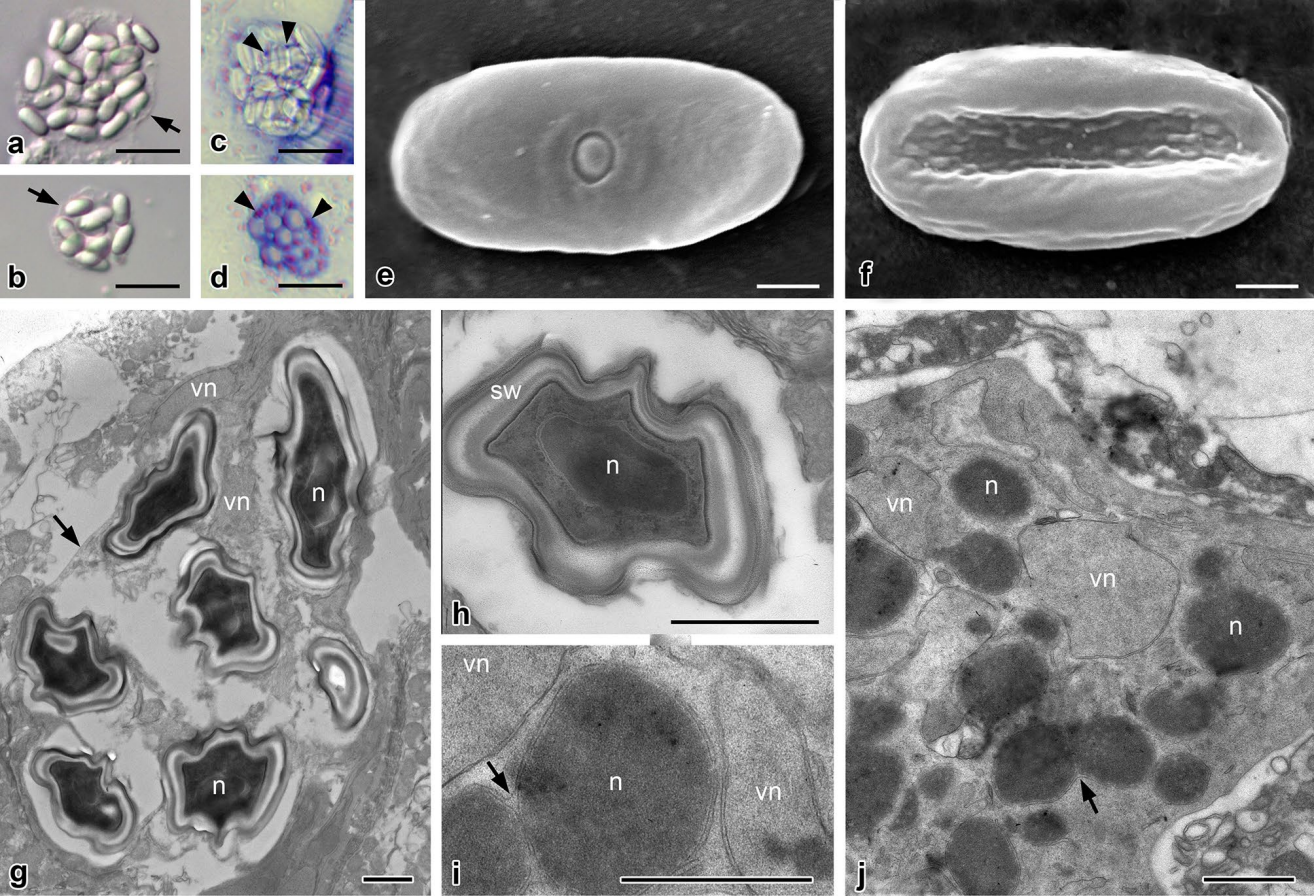
*Nephridiophaga javanicae* sp. nov. from *Elliptorhina javanica*. (**a**,**b**) Sporogenic plasmodia including different numbers of mature spores. Arrows point to plasma membranes of the plasmodia. (**c**,**d**) Giemsa staining reveals vegetative nuclei (arrowheads) of sporogenic plasmodia. (**e**,**f**) Scanning electron micrographs of dorsal (**e**) and ventral (**f**) side of flattened, mature spores. Dorsal side with central cap of spore opening. (**g**–**j**) Ultra-thin sections. (**g**) Sporogenic plasmodium with electron-dense mature spores and vegetative nuclei (vn). Spores contain one nucleus (n). Arrow points to plasma membrane of plasmodium. (**h**) Mature spore in cross-section. Spore wall (sw) consists of several layers. (**i**,**j**) Vegetative plasmodia with vegetative electron-light nuclei (vn) and prospective electron-dense spore nuclei (n). Arrows point to division or fusion of electron-dense nuclei (note outer membrane surrounding both nuclei). Scale bars: (a–d) = 10 µm, (e–j) = 1 µm.

Life cycle stages and morphological features of the two *Nephridiophaga* species from *Eublaberus posticus* (*Nephridiophaga postici*; Fig. 1) and *Elliptorhina javanica* (*Nephridiophaga javanicae*; Fig. 2) resemble each other. Developmental stages occur in the lumen of the Malpighian tubules (Fig. 1a). The most prominent stages are the sporogenic plasmodia, which include different numbers of spores (Figs.1a,b,d, 2a–d). Vegetative plasmodia can be recognised in Giemsa stained smears by the possession of multiple nuclei (Fig. 1e,f). The mostly spherical, sometimes elongated sporogenic plasmodia of *N. postici* measure 16.5–34.4 × 13.6–22.0 µm (mean 23.9 × 18.1 µm; *n* = 9) and contain 11–38 (mean 22; *n* = 21) spores. The sporogenic plasmodia of *N. javanicae* measure 14.2–22.7 × 12.9–20.3 µm (mean 18.8 × 16.5 µm; *n* = 22) and include 11–21 (mean 15.6; *n* = 22) spores. Between the spores, residual vegetative nuclei of the mother plasmodia are visible (Figs. 1d, 2c,d). Single mature spores are flattened, oval-shaped (Fig. 1c), and measure 5.0–7.0 × 2.6–3.8 µm (mean 5.9 × 3.2 µm; *n* = 52) in *N. postici* and 5.3–7.1 × 2.7–3.8 µm (mean 6.4 × 3.3 µm; *n* = 42) in *N. javanicae*.

Scanning electron microscopy revealed flattened spores with a thickened rim (Figs. 1g,h, 2e,f). One side possesses a small, central cap (spore opening; Figs. 1g, 2e; the dorsal side according to Radek and Herth^8^) while the other (ventral) side misses such a structure but shows tiny bulges. Longitudinal folds may occur as preparation artifact during drying (Figs. 1g, 2f). Between the electron-dense mature spores of sporogenic plasmodia, residual vegetative nuclei are seen in ultra-thin sections (Fig. 2g). Mature spores contain one central nucleus (Fig. 2g, h). The spore walls are thick at the rim and thinner at the flat sides and the spore opening (Fig. 1j). They consist of several layers (Figs. 1i,j, 2g,h); in addition to outer and inner biomembranes, the outer and inner layers of the wall are electron-dense and the zone in-between has a moderate electron density. Before visible spores are formed within a plasmodium, two types of nuclei develop, which differ by electron density (Fig. 2i,j). Since the residual vegetative nuclei in mature sporogenic plasmodia are electron-light (Fig. 2g), the electron-light nuclei in young sporogenic plasmodia (Fig. 2i,j) most likely also represent vegetative nuclei while the dense forms represent the future spore nuclei.

Whereas the described morphological characteristics of *N. postici* and *N. javanicae* were less discriminative, our molecular phylogenetic analyses (below) confirmed that they are two distinct species.

### Affiliation of nephridiophagids to Chytridiomycota

**Figure 3:**
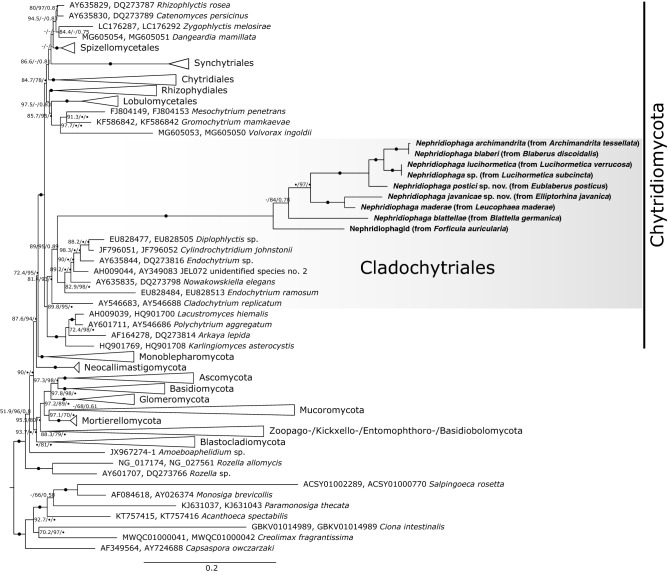
Phylogenetic tree inferred from a concatenated alignment of SSU and LSU rRNA genes under the GTR + F + I + G4 model. Branch support is given by SH-aLRT/UFBoot2/Bayesian posterior probabilities. Black circles indicate support values ≥ 99% or ≥ 0.9, and dashes indicate values < 50% or < 0.5. Black circles at branches show ≥ 99% and ≥ 0.9 support in all analyses. Sequences obtained in this study are marked in bold. Note, for nephridiophagids obtained from *Lucihormetica* spp. and *Archimandrita tessellata* only SSU rRNA gene sequences have been used for tree inference. See Supplementary Data for taxa of collapsed clades.

To place nephridiophagids within the fungal tree of life, we incorporated all major fungal groups in our analyses. The phylogenetic tree (Fig. 3) inferred from a concatenated alignment of SSU and LSU rRNA genes reveals overall good support for the diverse phyla (for a current overview, see Wijayawardene et al.^24^). Nephridiophagids fall into the phylum Chytridiomycota, branching as sister to species that belong to the Cladochytriales (support 89%, 95%, 0.89; see Fig. 3). This topology is also supported by the approximately unbiased test (*p* = 1), whereas alternative hypotheses of a sister relationship of nephridiophagids to (i) Mucoro-/Mortierellomycota, (ii) Zoopago-/Kickxello-/Entomophthoro-/Basidiobolomycota, and (iii) *Rozella* are all rejected (*p* = 0). These alternative topologies were tested as an affiliation of nephridiophagids to the—meanwhile as paraphyletic classified—Zygomycota or to the Rozellomycota has been hypothesised elsewhere (without statistical support though)^16,25^.

Monophyly of nephridiophagids was fully supported in all analyses. In addition to the here two formally described species, *Nephridiophaga postici* and *Nephridiophaga javanicae*, sequence data was obtained from further, so far only morphologically characterised nephridiophagids. They all branch with high support in close proximity to the only three *Nephridiophaga* species represented by both molecular and morphological data (*N. blattellae*, *N. blaberi*, and *N. maderae*)^4,8,9,17,26^, confirming their assignment to the genus *Nephridiophaga*. The only exception from this is the nephridiophagid isolated from the European earwig *Forficula auricularia*, which is more distantly related (~ 8% SSU rRNA gene sequence divergence) and branches as sister to the *Nephridiophaga* cluster (Fig. 3).

### Host specificity

**Figure 4:**
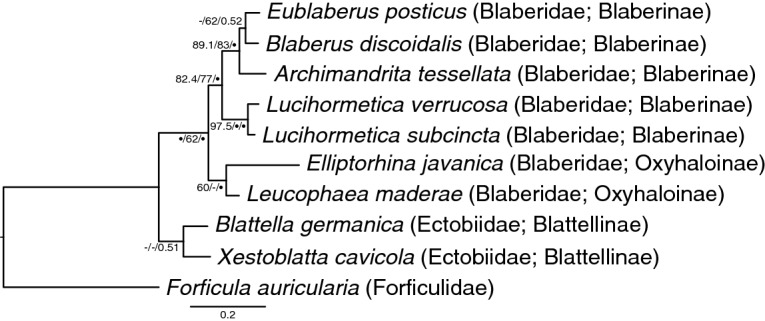
Phylogenetic tree of the host taxa inferred from the deduced amino acid alignment of the mitochondrial cytochrome *c* oxidase II. The topology is based on maximum-likelihood and Bayesian analyses (see “Methods”), and branch support is given by SH-aLRT/UFBoot2/Bayesian posterior probabilities. Black circles indicate support values ≥ 99% or ≥ 0.9, and dashes indicate values < 50% or < 0.5. The amino acid sequence of *Xestoblatta cavicola* has been added to sustain the sister-relationship of Blattellinae and Blaberidae^18^. The tree is rooted on *Forficula auricularia* (Dermaptera).

To check for strict host specificity and the resulting potential for cospeciation of the host insects and their fungal parasites (i.e., for congruency of their phylogenies), we reconstructed the phylogenetic relationships between the insects that harbor nephridiophagids (Fig. 4). Despite the fact that several nodes of the host tree remained rather inconclusive, it is conspicuous that the topology of the early-branching lineages (*F. auricularia*, *B. germanica*, *L. maderae*, and *E. javanica*) mirrors the topology of their respective parasites (see Figs. 3 and 4). However, even without further testing a strict host specificity or even cospeciation of the two partners in general cannot be confirmed, not only because of the distinct branching of *E. posticus* in a more apical clade (compare Figs. 3 and 4) but also because we detected virtually identical *Nephridiophaga* sequences obtained from both *A. tessellata* and *B. discoidalis* (Fig. 3; > 99.8% SSU rRNA gene sequence identity; ITS/LSU data was not available for *Nephridiophaga archimandrita*).

## Discussion

Although the fungal character of nephridiophagids has been revealed more than 15 years ago^16^, their phylogenetic affiliation to a certain fungal phylum remained enigmatic. Based on SSU rRNA gene sequence analyses, relationships either to the Zygomycota^16,27^ or close to the root of the fungal kingdom^17^ have been proposed. In this study, we increased the phylogenetic signal by analysing both the SSU and LSU rRNA gene sequences of numerous, partly newly described species. Our results show that nephridiophagids belong to the phylum Chytridiomycota (also known as “chytrids”).

### Phylogenetic assignment

Nephridiophagids show only a few morphological characteristics hampering their classification based on microscopic studies. The here newly described species, *Nephridiophaga postici* and *Nephridiophaga javanicae*, resembled the general morphology of other *Nephridiophaga* species concerning the shape and size of spores and vegetative and sporogenic plasmodia, as well as the number of spores in a sporogenic plasmodium (summarised by Radek et al.^7^). Thus, due to transitions between these features among different nephridiophagid species and the fact that the same species can infect two different host species (see below), we propose that molecular markers will additionally be considered in future classifications.

Our phylogenetic analyses of a concatenated SSU/LSU rRNA gene alignment corroborated the novel species character of *N. postici* and *N. javanicae* and, moreover, allowed an unambiguous assignment of nephridiophagids to the Chytridiomycota. The consideration of the LSU rRNA gene has previously been shown to boost the phylogenetic power in tree inferences, allowing to untangle the diversification not only of early-branching fungi^28–32^ but in addition with the SSU also of other clades such as dinoflagellates or even higher-ranking and deep-branching groups^33,34^. Here, nephridiophagids fell into the fully supported Chytridiomycota clade. Their further affiliation to the Cladochytriales remains less definite as the node in question did not yield maximal statistical support (although SH-aLRT, UFBoot2, and Bayesian PP were all in a range that typically confirms a certain clade^35–37^; Fig. 3). Note that for the used ultrafast bootstraps, which provide more unbiased branch supports than standard bootstraps, a reliability threshold of ≥ 95% is recommended^37^. The long stem of the nephridiophagids, however, does not automatically reflect phylogenetic distance to the other Cladochytriales species but rather underlines their rapidly-evolving character, which is frequently observed in parasitic eukaryotes^38–41^. Also, the parasitic life style and the lack of flagellated zoospores in nephridiophagids do not necessarily justify an exclusion from the Cladochytriales. Though members of this order show a characteristic zoospore ultrastructure^42^ and inhabit aquatic habitats and moist soils where they grow on decaying plant material^42,43^, all different kind of aquatic and terrestric environments and life styles (saprophytic, parasitic, saprophytic/parasitic) have been documented within numerous unrelated chytrid orders [e.g.,^44–49^, indicating that parasitism has evolved several times independently in this group. Similarly, the absence of flagellated zoospores is likely phylogenetically less informative but denotes a secondary loss in adaptation to an endoparasitic lifestyle and a passive spore transmission via the oral uptake of feces. Although most parasitic lineages (infecting algae, land plants, fungi or reptiles) still possess free-swimming zoospores that settle on a potential host, losses of flagella have been reported for some non-related groups within chytrids and also monoblepharomycotes and other early-branching fungi such as microsporidians—especially when the life cycle is endoparasitic in all stages^30,50–52^.

### Host specificity

Nephridiophagids have long been assumed to be host-specific. Indeed, feeding spores from one cockroach species to another nephridiophage-free cockroach species did not lead to new infections in transmission experiments^9^. In these studies, however, closely related hosts (belonging to the same family) were not tested. Due to the shared and inconspicuous morphology of nephridiophagids, addressing the question of host specificity solely based on microscopic studies is difficult, but so far, molecular markers (SSU rRNA gene sequences) were known only for three *Nephridiophaga* species^16,17^. Our molecular phylogenetic studies allowed the detection of the same *Nephridiophaga* phylotype in the cockroaches *Archimandrita tessellata* and *Blaberus discoidalis*. The nephridiophagids of these two hosts have previously been treated as separate species described as *Nephridiophaga archimandrita* (lacking molecular data^7^) and *Nephridiophaga blaberi* (with published SSU rRNA gene sequence^4,17^). In this context, it is noteworthy that both Fabel et al.^4^ and Radek et al.^17^ misidentified the host of *N. blaberi* as *Blaberus craniifer*. Here, we tested the identity of this cockroach (from the same source mentioned in the two studies) based on its mitochondrial COII gene sequence, and we correct as follows: the host of *N. blaberi* is *B. discoidalis* (occasionally named as “false death’s head cockroach”) and not the similar looking *B. craniifer* (death’s head cockroach). Considering their identical SSU rRNA gene sequences, we further suggest to synonymise the described species *N. archimandrita* and *N. blaberi* upon the original host can be identified.

The presence of the same nephridiophagid phylotype in two different cockroach species shows that a transmission is generally possible—at least temporarily and between closely related species. Here, both host species were affiliated to the subfamily Blaberinae. We hypothesise that the observed horizontal transfer is a consequence of maintaining the cockroaches in the same cultural area at our institute and at the German Environment Agency (from where they were obtained) for many years. Although not tested, taking into account the high numbers of spores and sporangia found in microscopic observations, we believe that the nephridiophagid phylotype in question became a permanent parasite of both cockroach species, *A. tessellata* and *B. discoidalis*. Whether one of the two hosts was primarily free of *Nephridiophaga* infection or whether one *Nephridiophaga* species has been substituted by another is currently unclear. A release of selection pressure and lower microbiome diversity in laboratory animals may facilitate the colonisation of opportunistic infections. Thus, it is speculative to what extant horizontal nephridiophagid transfer can be observed outside the laboratory. Partial congruence of the phylogenetic trees of hosts and parasites implies that parasite exchange did not happen between the early-branching, more distantly-related host insects (Figs. 3 and 4). However, if this observation is simply caused by their different distribution needs to be investigated in future studies. The parasites’ life cycle, which includes a release of spores with the host feces and new infection by their oral ingestion, enables an easy interspecific transfer. Yet, if this then leads to a permanent infection presumably depends on the degree to which the parasite evolved host-specific adaptations over time (as shown for many other symbiont/host systems^19–23,53,54^). Our observations give rise to believe that nephridiophagids are less host-specific than generally assumed excluding a strict cospeciation with their host insects. This is also in line with the scattered presence of these parasites even within a certain family of diverse insects (Supplementary Table S1).

### General notes

To date, most *Nephridiophaga* species have only been described by a few morphological features such as spore size, the number of spores per sporogenic plasmodium, and the habitat for spore formation (in the lumen or exceptionally within the epithelial cells of the Malpighian tubules)^4^. Since in general, the stages of different species look very similar, the presumed host specificity was traditionally used as further criterion for delimitating new species—a distinguishing trait that is no longer recommended by our findings. Here, we described two new *Nephridiophaga* species based on both, morphology and molecular phylogeny. SSU and LSU rRNA gene sequences have been obtained from several further nephridiophagids, among them a species from an earwig, which has morphologically been described as *Nephridiophaga forficulae*^6^ but possibly represents a further genus of nephridiophagids. We did not find the type species *Nephridiophaga apis*, but considering the genetic distance between *N. forficulae* and cockroach-infecting nephridiophagids, it is possible that *N. apis* is even more distantly related to most of the described nephridiophagids—a discovery that would challenge their assignment to the genus *Nephridiophaga*.

Although the described methods enabled us to obtain sequence data, which allowed an assignment of nephridiophagids to the Chytridiomycota, it needs to be mentioned that the protocol will require some modifications. Whereas it worked fine for host individuals that were highly infected by nephridiophagids, only a few reads were obtained for less infected individuals, hindering the generation of reliable consensus sequences. We tested annealing temperatures of 55 °C and 57 °C for the long-range PCRs. Yet, for both settings the majority of reads belonged either to the host insect or other fungi such as yeasts. As the primers span the SSU, ITS1, 5.8S, ITS2, and a long part of the LSU rRNA gene, they are not too easy to replace. We therefore suggest trying even higher annealing temperatures and/or—where possible—to increase the ratio of target cells by for example micromanipulation or centrifugation in future studies. In combination with genome amplification or other techniques, the latter could also facilitate the sequencing of numerous further genes that can be used for tree inference. An approach, which will become favourable as soon as more diverse chytrid genomes/transcriptomes become publicly available.

### Taxonomy

#### *Nephridiophaga postici *Strassert and Radek sp. nov.

*Registration identifier* MycoBank No. MB837477.

*Diagnosis* Flattened, oval to elongate, uninucleate spores measuring 5.0–7.0 × 2.6–3.8 μm (mean 5.9 × 3.2 µm; n = 52) in fresh preparations. 11–38 (mean 22; n = 21) spores per sporogenic plasmodium. Sporogenic plasmodia mostly spherical, sometimes elongated, measuring 16.5–34.4 × 13.6–22.0 µm (mean 23.9 × 18.1 µm; n = 9).

*Holotype* A Giemsa-stained smear with slide number 2020/14 was deposited in the Upper Austrian Museum in Linz, Austria.

*Distribution/host locality* Cockroach hosts are cultured at the insectarium of Aquarium Berlin, Berlin, Germany. *E. posticus* is native to Central and South America.

*Ecology* Infection of the host by oral ingestion of spores. Life cycle stages develop in the Malpighian tubules. Spores released via the feces.

*Etymology and host* Named after its host *Eublaberus posticus* Erichson, 1848.

*Gene sequence* rDNA operon acc. no. MW018148.

#### *Nephridiophaga javanicae* Strassert and Radek sp. nov.

*Registration identifier* MycoBank No. MB837478.

*Diagnosis* Flattened, oval to elongate, uninucleate spores measuring 5.3–7.1 × 2.7–3.8 μm (mean 6.4 × 3.3 µm; n = 42) in fresh preparations. 11–21 (mean 15.6; n = 22) spores per sporogenic plasmodium. Sporogenic plasmodia mostly spherical, measuring 14.2–22.7 × 12.9–20.3 µm (mean 18.8 × 16.5 µm; n = 22).

*Holotype* A Giemsa-stained smear with slide number 2020/13 was deposited in the Upper Austrian Museum in Linz, Austria.

*Distribution/host locality* Cockroach hosts are cultured at the insectarium of Aquarium Berlin, Berlin, Germany; naturally occurring in Madagascar.

*Ecology* Infection of the host by oral ingestion of spores. Life cycle stages develop in the Malpighian tubules. Spores released via the feces.

*Etymology and host* Named after its host *Elliptorhina javanica* Hanitsch, 1930.

*Gene sequence* rDNA operon acc. no. MW018146.

## Methods

### Sampling

The following insects were used in this study: *Archimandrita tessellata* (peppered cockroach), *Blaberus discoidalis* (discoid cockroach), *Blattella germanica* (German cockroach), *Elliptorhina javanica* (Halloween hisser), *Eublaberus posticus* (orange head cockroach), *Leucophaea maderae* (Madeira cockroach), *Lucihormetica subcincta* (glow spot cockroach), *Lucihormetica verrucosa* (warty glow spot cockroach), and *Forficula auricularia* (European earwig). A list of the numerous further insects microscopically checked for parasites with nephridiophagid morphology is given in Supplementary Table S1. Prior to preparation, individuals were either killed by freezing or, for morphological investigations, by crushing the head. Parts of the Malpighian tubules were removed, transferred into Ringer solution (Sigma-Aldrich), and checked for nephridiophagids by light microscopy. In case of infection, the remaining parts of tubules were stored in RNA*later* stabilisation solution (Sigma-Aldrich) for molecular phylogenetic analyses. Material from further animals was processed for morphological studies.

### Morphological analyses

Disrupted Malpighian tubules were smeared on microscope slides, air-dried, fixed in methanol (5 min), and stained with Giemsa solution (Accustain, Sigma-Aldrich; 45 min in a 1:10 dilution). Dried smears were embedded in Entellan (Merck). For scanning electron microscopy, cover glasses with smears of infected Malpighian tubules were air-dried and coated with gold particles using a Baltec SCD 040 sputter device. Micrographs were taken with a Quanta 200 electron microscope (FEI Company). For transmission electron microscopy, samples were fixed for several days with 2.5% glutaraldehyde in 0.1 M cacodylate buffer at 4 °C, rinsed with cacodylate buffer, and post-fixed for 1.5 h with reduced OsO_4_ at room temperature without darkening (fresh 1:1 mixture of 2% OsO_4_ and 3% K_4_[Fe(CN)_6_]). The samples were then rinsed with distilled water, and after dehydration in a series of ethanol (15 min each in 30%, 50%, 70%, 90%, 100%, 3 × 100% water free), they were embedded in Spurr’s resin^55^. Ultrathin sections were stained with saturated aqueous uranyl acetate for 30 min in the dark, followed by Reynolds’ lead citrate^56^ for 5 min. During the latter, sodium hydroxide pellets were put next to the grids in order to remove CO_2_ from the atmosphere and thus prevent precipitation of lead carbonate. The sections were investigated using a Philips CM 120 BioTwin electron microscope.

### DNA extraction and long-range PCRs

To avoid DNA shearing, samples were carefully disrupted by only one freeze-thawing cycle followed by bead-beating with two steel beads (diameter: 3 mm) in a 2 mL tube using a Retsch MM 400 Mixer Mill (45 s at 30 1/s). DNA was extracted with the DNeasy Plant Mini Kit (Qiagen) following the manufacturer’s instructions with two exceptions: (i) after the initial incubation in Buffer AP1 + RNase for 10 min at 65 °C, lysates were kept at room temperature for another 20 min, (ii) lower volumes of Buffer AE were used for elution. For PCR-amplification of the SSU rRNA, ITS1, 5.8S rRNA, ITS2, and most of the LSU rRNA gene, the Herculase II Fusion DNA Polymerase (Agilent Technologies) was used together with the primers NS1short and RCA95m^57^, each prolonged at the 5′-end with Oxford Nanopore Universal Tags (TTT CTG TTG GTG CTG ATA TTG C-NS1short, ACT TGC CTG TCG CTC TAT CTT C-RCA95m). The PCR started with a denaturing step at 95 °C for 4 min, followed by 36 cycles at 95 °C for 25 s, 55 (or 57) °C for 25 s and 70 °C for 4 min, and a final extension step at 70 °C for 6 min. PCR products were then used as template for another PCR with 13 cycles and an annealing temperature of 62 °C but otherwise identical settings. The primers in this second PCR were replaced by sample-specific barcodes.

### Library preparation and Nanopore sequencing

Products of the barcoding PCR were purified using the Agencourt AMPure XP Kit (Beckman Coulter) and quantified with both Qubit (Invitrogen) and NanoDrop (Thermo Fisher). For each sequencing run on a flow cell, four to six samples (not all part of this study) were pooled in an equimolar way yielding a total of 1 µg DNA. Library preparation comprised a DNA end repair, adapter ligation, and intermediate purification steps, and was carried out according to the 1D Sequencing SQK-LSK108 protocol (Oxford Nanopore Technologies). Samples were sequenced with a MinION equipped with FLO-MIN106 flow cells (R9.4; Oxford Nanopore Technologies). High-accuracy base calling was employed using Guppy implemented in the MinKNOW software (Oxford Nanopore Technologies).

### Long-reads processing

Reads shorter than 3500 bp, longer than 8000 bp and with more than 10 homopolymers were discarded using mothur v. 1.43.0^58^. They were then classified with the naive Bayesian classifier^59^ implemented in mothur using an in-house database containing Nephridiophagidae and an 80% confidence threshold. Subsequently, target sequences were extracted and demultiplexed using Flexbar^60^, aligned with MAFFT 7^61^, and clustered employing the opticlust option in mothur. Final consensus sequences were then generated with Consension (https://microbiology.se/software/consension/).

### SSU rDNA sequencing and host COII sequencing

For *A. tessellata*, *L. subcincta*, and *L. verrucosa*, the number of *Nephridiophaga* reads obtained by Nanopore sequencing was too low (< 5) to create reliable consensus sequences. Here, the SSU rDNA was amplified using the KAPA2G Fast HotStart ReadyMix and universal eukaryote primers^62^ in combination with newly designed nephridiophagid specific primers; according to Radek et al.^17^ but slightly modified due to a found mismatch: Neph_F, CAG TTG GGG GCG TYA GTA TT and Neph_R, AAT ACT RAC GCC CCC AAC TG. The PCR started with a denaturing step at 94 °C for 5 min, followed by 35 cycles at 94 °C for 1 min, 59 °C for 1 min, and 72 °C for 1 min, and a final extension step at 72 °C for 10 min. PCR products were cleaned and sequenced at LGC, Biosearch Technologies.

The mitochondrial COII genes of the host insects were amplified with the primers Mod A-tLeu and B-tLys2 (CAG ATA AGT GCA TTG GAT TT and GTT TAA GAG ACC AGT ACT TG, respectively; modified from Liu and Beckenbach^63^) using the KAPA2G Fast HotStart ReadyMix. The PCR started with a denaturing step at 94 °C for 3 min, followed by 35 cycles at 94 °C for 30 s, 56 °C for 45 s, and 72 °C for 90 s, and a final extension step at 72 °C for 7 min. PCR products were cleaned and sequenced at LGC, Biosearch Technologies.

### Phylogenetic analyses

Newly obtained nephridiophagid SSU and LSU rRNA gene sequences were aligned together with representatives of major fungal groups using MAFFT L-INS-i v. 7.055b^64^ (Supplementary Data) and filtered with trimAL v. 1.2rev59^65^ using a gap threshold of 0.3 and a similarity threshold of 0.001. Sequences were then concatenated with SeqKit v. 0.11.0^66^. A maximum-likelihood tree was inferred from the concatenated alignment with IQ-TREE v. 1.6.12^67^ employing the best-fitting model GTR + F + I + G4 (determined with ModelFinder^68^). Copies of the obtained tree were manually edited to test alternative topologies using the approximately unbiased test^69^: 1) nephridiophagids sister to Cladochytriales (original tree) *versus* nephridiophagids sister to the Mucoro-/Mortierellomycota clade, 2) nephridiophagids sister to Cladochytriales *versus* nephridiophagids sister to the Zoopago-/Kickxello-/Entomophthoro-/Basidiobolomycota clade, 3) nephridiophagids sister to Cladochytriales *versus* nephridiophagids sister to *Rozella*. Branch support was assed using ultrafast bootstrap approximation^37^ (UFBoot2; 1,000 replicates) and SH-like approximate likelihood ratio test (SH-aLRT)^35^ (1,000 replicates). Bayesian analysis was inferred with PhyloBayes-MPI v. 1.8^36^ using the GTR model and four categories for the discrete gamma distribution (53,700 generations; burnin 6000). Convergence of two independent Markov Chain Monte Carlo (MCMC) chains was tested with bpcomp and confirmed with maxdiff reaching 0.05.

COII nucleic acid sequences of the hosts were translated to amino acid sequences with EMBOSS Transeq^70^, aligned with MAFFT L-INS-I (Supplementary Data), and filtered with trimAl (-automated1 flag employed). A phylogenetic tree was reconstructed using IQ-TREE under the best-fitting mitochondrial Metazoa protein model mtMet^71^ + G4, and node support was inferred with ultrafast bootstrap approximation (UFBoot2; 1,000 replicates) and SH-aLRT (1000 replicates). A second tree based on Bayesian analysis was built with PhyloBayes-MPI (model -dgam 4 -cat -gtr). Convergence of four independent MCMC chains (128,000 generations; burnin 14,000) was confirmed with maxdiff reaching 0.006.

## Supplementary Information

Supplementary Information.

Supplementary Table S1.

## Data Availability

Sequence data are available under Acc. Nos. MT993857–MT993859 and MW018144–MW018149. All other data needed to evaluate the conclusions in the paper are present in the paper and the Supplementary Information.
